# Increased incidence of ectopic pregnancy after *in vitro* fertilization in women with decreased ovarian reserve

**DOI:** 10.18632/oncotarget.14679

**Published:** 2017-01-16

**Authors:** Shengli Lin, Rui Yang, Hongbin Chi, Ying Lian, Jiejing Wang, Shuo Huang, Cuiling Lu, Ping Liu, Jie Qiao

**Affiliations:** ^1^ Reproductive Medical Center, Department of Obstetrics and Gynecology, Peking University Third Hospital, Beijing, China

**Keywords:** IVF, decreased ovarian reserve, ectopic pregnancy

## Abstract

The incidence of ectopic pregnancy after assisted reproductive technology is increased approximately 2.5–5-fold compared with natural conceptions.

Strategies were used to decrease the incidence of ectopic pregnancy, but ectopic pregnancy still occurs. In the present study, women were selected with decreased ovarian reserve (defined as FSH > 10 IU/L) aged 20 to 38 years who underwent IVF-ET between 2009 and 2014. These 2,061 women were age-matched with an equal number of women with normal ovarian reserve (defined as FSH ≤ 10 IU/L). During cycles following fresh embryo transfer, 93 patients were diagnosed with ectopic pregnancy. The incidence of ectopic pregnancy in clinical pregnancies was significantly higher in the decreased ovarian reserve than in the normal ovarian reserve group (5.51% vs. 2.99%). After adjusting for confounding factors, the incidence of ectopic pregnancy was significantly associated with decreased ovarian reserve. Our results showed that decreased ovarian reserve is an independent risk factor for ectopic pregnancy after *in vitro* fertilization-embryo transfer.

## INTRODUCTION

The incidence of ectopic pregnancy (EP) is 1.0%–2.0% of all pregnancies, the diagnosis can be difficult due to unusual locations and consequently it carries a serious health risk to pregnant women [1, 2]. EP accounts for 31.9 pregnancy-related deaths per 100,000 pregnancies in the United States [3]. Primary risk factors for EP include smoking, intrauterine devices, previous EP, pelvic inflammatory disease, and tubal factors [4]. Moreover, the use of assisted reproductive technology (ART) increases the incidence of EP. Compared with natural conception, the EP rate is approximately 2.5–5-fold higher following *in vitro* fertilization-embryo transfer (IVF-ET), although this rate has been declining [5]. Studies have shown that the use of assisted hatching, higher transfer volume, deep fundal transfer, day of embryo transfer, changes in endometrial receptivity, and multiple embryo transfer increase the incidence of EP [6, 7].

Women of the same age may possess different responses to ovarian stimulation, leading to a variation in the reproductive potential. Women of reproductive age with regular menses who respond poorly to ovarian stimulation or fecundity compared with others of the same age are defined as having decreased ovarian reserve (DOR) [8]. The prevalence of DOR is approximately 10% among infertile women and leads many to seek ART.

Several methods are available for detecting DOR. Follicle-stimulating hormone (FSH) is secreted by the gonadotrophs of the anterior pituitary gland and promotes follicle development. Serum FSH levels increase with age, and an early follicular phase serum FSH level > 10 IU/L is a predictor of DOR. In addition, anti-Mullerian hormone (AMH), the antral follicle count, ovarian volume, and the clomiphene citrate challenge test are also used to predict the ovarian reserve [9]. Women with DOR display decreased fertilization rates and increased blastocyst aneuploidy and miscarriage rates [10, 11].

Recent efforts have been made to predict the occurrence of EP prior to an IVF cycle, without defining a clear algorithm yet [12–14]. In the present study, we wondered if DOR can predict the occurrence of EP. To explore the issue, we compared EP rates between patients with DOR and those with normal ovarian reserve (NOR) following IVF-ET.

## RESULTS

**Figure 1:**
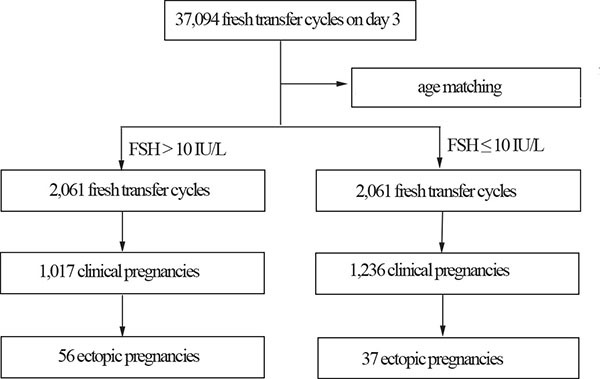
Patient selection flowchart

2,061 age-matched women were analyzed between DOR and NOR groups. As shown in Figure 1, the number of clinical pregnancies was 1,236 in the NOR group and 1,017 in the DOR group. The characteristics of the pregnant women are shown in Table 1. The average FSH level was significantly higher in the DOR than in the NOR group (12.64 ± 3.75 IU/L vs. 6.02 ± 2.15 IU/L). The maternal BMI and number of oocytes retrieved were lower in the DOR than in the NOR group, while the dose of gonadotrophin was higher in the DOR than in the NOR group. Other characteristics did not differ significantly between the two groups.

**Table 1:** Demographic and clinical characteristics of women who became pregnant after *in vitro* fertilization according to their ovarian reserve status

Characteristic	FSH ≤ 10 IU/L (*n* = 1,236 )	FSH > 10 IU/L (*n* = 1,017)	*P* value
FSH (IU/L)	6.02 ± 2.15	12.64 ± 3.75	< 0.001
Maternal age (years)	31.48 ± 3.23	31.58 ± 3.17	0.454
Paternal age (years)	33.31 ± 4.61	33.53 ± 4.54	0.256
Maternal BMI (kg/m^2^)	22.53 ± 3.31	21.53 ± 3.08	< 0.001
Paternal BMI (kg/m^2^)	25.38 ± 3.51	25.32 ± 3.91	0.711
Duration of subfertility (years)	4.67 ± 2.99	4.45 ± 2.95	0.089
Tubal factor	591 (47.82%)	467 (45.92%)	0.369
Primary subfertility	695 (56.23%)	598 (58.80%)	0.219
No. of oocytes retrieved	12.97 ± 5.74	8.99 ± 5.28	< 0.001
No. of embryos transferred	2.10 ± 0.38	2.07 ± 0.47	0.053
Cycles with ICSI	537 (43.45%)	410 (40.32%)	0.134
Dose of gonadotrophin (IU)	2664 ± 1187	3523 ± 1474	< 0.001
Previous tubal surgery	99 (8.01%)	76 (7.47%)	0.636
Previous ectopic pregnancy	98 (7.93%)	60 (5.90%)	0.061
Previous miscarriage	26 (2.10%)	17 (1.67%)	0.456

Notes: Continuous variables were compared using analysis of variance and categorical variables were evaluated using chi-square tests.

*P* values indicate statistically significant differences between the groups.

The clinical pregnancy rate was significantly higher in patients with NOR than in those with DOR (47.52% vs. 39.10%) (Table 2). During these pregnancies, 93 patients were diagnosed with EP, 37 in the NOR group and 56 in the DOR group, an incidence rate in clinical pregnancies significantly higher in the DOR versus the NOR group (5.51% vs. 2.99%, *P* = 0.003).

**Table 2:** Incidence of ectopic pregnancy after *in vitro* fertilization in women with or without decreased ovarian reserve

Characteristic	FSH ≤ 10 IU/L (*n* = 2061)	FSH > 10 IU/L (*n* = 2061)	*P* value
Maternal age (years)	31.79 ± 3.14	31.79 ± 3.14	_
Clinical pregnancy	1236 (47.52%)	1017 (39.10%)	< 0.001
EP	37	56	
EP per clinical pregnancy (%)	2.99	5.51	0.003

Notes: Data are presented as the numerical values and %; Categorical variables were evaluated using chi-square tests. *P* values indicate statistically significant differences between the groups.

After adjusting for confounding factors, DOR significantly increased the risk of EP (OR = 2.128, 95% CI 1.300–3.482, *P* = 0.003, with NOR as the reference group) (Table 3).

**Table 3:** Factors associated with ectopic pregnancy after *in vitro* fertilization in women with decreased compared with those with normal ovarian reserve

Parameters	*P* value	OR (95% CI)
DOR (versus no DOR)	0.003	2.128 (1.300–3.482)
Maternal age (years)	0.080	1.092 (0.990–1.204)
Paternal age (years)	0.112	0.945 (0.882–1.013)
Maternal BMI (kg/m^2^)	0.925	1.003 (0.936–1.075)
Paternal BMI (kg/m^2^)	0.154	0.956 (0.899–1.017)
Duration of subfertility (years)	0.876	0.994 (0.920–1.074)
Primary subfertility	0.997	0.999 (0.524–1.904)
No. of oocytes retrieved	0.763	1.006 (0.966–1.048)
No. of embryo transferred	0.168	1.423 (0.862–2.350)
Cycles with ICSI	0.300	1.275 (0.805–2.018)
Dose of gonadotrophin (IU)	0.457	1.000 (1.000–1.000)
Gravidity	0.506	1.102 (0.828–1.466)
Parity	0.177	0.371 (0.088–1.564)
Previous tubal surgery	0.515	1.303 (0.587–2.890)
Previous ectopic pregnancy	0.735	0.851 (0.334–2.171)
Previous miscarriage	0.429	1.678 (0.466–6.047)

Notes: Multivariate logistic regression analysis was used to assess risk factors.

## DISCUSSION

Our retrospective study showed that DOR is an independent risk factor for EP after IVF-ET. The EP rates in clinical pregnancies were significantly higher in women with DOR compared with those with NOR.

It is well known that ART leads to a higher incidence of EP compared with spontaneous conceptions, although to date, the underlying etiology remains unclear. The study of IVF-ET procedures provides an important opportunity to explore embryo implantation processes that cannot be observed in spontaneous conception. Large, population-based studies have shown that tubal infertility, the number of embryos transferred, length of embryo culture and some metabolic pathways and proteins are significantly associated with the incidence of EP [12–14]. With a reduction in tubal factor infertility, transferring fewer embryos, and extended embryo culture, EP rates following IVF-ET have progressively decreased [15]. However, EP continues to occur. Out of 2,601 age-matched IVF cycles evaluated in the present study, 93 EPs were identified. The EP rate of 2.99% of clinical pregnancies in the NOR group is similar to that reported previously [16], but we observed a significantly higher rate of 5.51% in the DOR group. Multivariate logistic regression analysis was performed to determine the relationship between DOR and EP. After adjusting for confounding factors, the incidence of ectopic pregnancy was significantly associated with DOR.

Oocyte development depends on the follicular microenvironment. The oocyte promotes the proliferation and differentiation of cumulus cells by means of oocyte-secreted factors. Conversely, cumulus cells support the growth and maturation of the oocyte through the transfer of growth factors, secondary messengers, and nutrients [17]. The disruption between cumulus cells and oocyte inhibits oocyte development. Microarray-based gene expression analysis has shown that CXXC5 (CXXC finger protein 5) was significantly downregulated in corona radiata cells from patients with DOR [18]. CXXC5 can interact with and inhibit the wingless type MMTV integration/β-catenin signaling pathway expressed in human trophoblastic cells [19, 20]. Mainly localized to cell-cell junctions, β-catenin plays an important role in intercellular adhesion, cell polarity, and architecture by binding to E-cadherin [21, 22]. Compared with non-pregnant tubal tissues, immunohistochemistry demonstrated that β-catenin was strongly expressed at tubal implantation sites in EPs following IVF-ET [23]. It is possible that the downregulation of CXXC5 increases expression of β-catenin, leading to a higher risk of EP in DOR. However, the underlying mechanisms need additional research.

In the present study, our analysis was limited by the retrospective nature of the data. The parameters of maternal smoking and pelvic inflammatory diseases were not acquired in the present study, so we could not observe the effect of maternal smoking and pelvic inflammatory diseases on incidence of EP. In addition, serum AMH levels were not obtained during the study period in our center. Further research will be needed to analyze the association between EP and DOR according to AMH levels. Several studies have recently tried to identify predictors of EP before the initiation of an IVF cycle; we think that DOR markers could be added to a future predictor algorithm. Preconception period has been recently recognized as a strategic time influencing subsequent pregnancy outcome [24]. IVF offers us the prime opportunity to work on the preconceptions period and we provide evidence supporting the importance on identification of DOR patients.

In conclusion, the present study showed that DOR increases the risk of EP after IVF-ET. More research needs to be conducted to explore the underlying mechanisms accounting for this increased risk.

## MATERIALS AND METHODS

### Patients

This retrospective study was approved by the ethics committee of Peking University Third Hospital. For this study, early follicular phase serum levels of FSH were determined using a commercial immunoassay system (Siemens, Immulite 2000 FSH). Women were selected with DOR (defined as FSH > 10 IU/L) aged 20 to 38 years who underwent IVF-ET between 2009 and 2014. These 2,061 women were age-matched with an equal number of women with NOR (defined as FSH ≤10 IU/L). Patients were excluded if they received preimplantation genetic diagnosis (PGD) or required oocytes donation.

Patients participating in fresh cycles underwent controlled ovarian hyperstimulation with a GnRH agonist or GnRH antagonist protocol as described previously [25]. Ovarian follicle development was monitored on the basis of serum estradiol (E2) levels and transvaginal ultrasonographic measurements. When at least one follicle reached a mean diameter of 18 mm and the E2 level exceeded 500 pg/ml, 10,000 units of urinary hCG (Serono, Aubonne, Switzerland) were administered before ultrasonography-guided oocyte retrieval. Regular luteal support was given with either 60 mg progesterone intramuscular injection or vaginal progesterone (Crinone 8% vaginal gel, Merck-Serono) daily.

### Laboratory protocols

IVF and ICSI were performed according to routine laboratory insemination procedures on the day of oocyte retrieval. The presence of two pronuclei was observed 17 ± 1 h after insemination or injection, and the zygotes were then cultured in 25 μl of pre-equilibrated cleavage medium droplets. The embryos were cultured in incubators at 37°C under 5% or 6% CO_2_. The morphology of embryos was evaluated 68 ± 1 h after insemination with respect to cell number, fragmentation, and symmetry. The number of embryos transferred was determined based on patient age, number of IVF cycles, and embryo quality. Clinical pregnancy was diagnosed by the detection of a gestational sac with a fetal heart beat at 7 weeks of gestation. EP was defined as sonographic visualization of a gestational sac outside the uterine endometrium or an empty uterine cavity accompanied by rising hCG levels [26]. The incidence of EP in the DOR and NOR groups were compared.

### Statistical analysis

Statistical analyses were conducted using SPSS software (version 16.0). Results for continuous data are reported as the mean ± standard deviation and categorical variables are reported as frequencies (%). Associations between DOR and EP were assessed using chi-square tests. Multivariate logistic regression analysis was used to assess the association between DOR and EP after adjusting for maternal age, paternal age, maternal and paternal BMI, duration of subfertility, subfertility type, number of oocytes retrieved, number of transferred embryos, fertilization method (IVF or ICSI), and dose of gonadotrophin, gravidity, parity, previous tubal surgery, previous ectopic pregnancy and previous miscarriage.

## Abbreviations

ART: assisted reproductive technology
IVF-ET: *in vitro* fertilization-embryo transfer
DOR: decreased ovarian reserve
NOR: normal ovarian reserve
EP: ectopic pregnancy
FSH: Follicle-stimulating hormone
AMH: Mullerian hormone
ICSI: intra-cytoplasmic sperm injection
